# Body composition reference values in Singaporean adults using dual-energy X-ray absorptiometry—The Yishun study

**DOI:** 10.1371/journal.pone.0276434

**Published:** 2022-10-21

**Authors:** BaoLin Pauline Soh, Shuen Yee Lee, Wai Yin Wong, Benedict Wei Jun Pang, Lay Khoon Lau, Khalid Abdul Jabbar, Wei Ting Seah, Kexun Kenneth Chen, Sivasubramanian Srinivasan, Tze Pin Ng, Shiou-Liang Wee

**Affiliations:** 1 Health and Social Sciences Cluster, Singapore Institute of Technology, Singapore, Singapore; 2 Department of Nuclear Medicine and Molecular Imaging, Singapore General Hospital, Singapore, Singapore; 3 Duke-NUS Medical School, Singapore, Singapore; 4 Geriatric Education and Research Institute (GERI), Singapore, Singapore; 5 Diagnostic Radiology, Khoo Teck Puat Hospital, Singapore, Singapore; 6 Department of Psychological Medicine, National University of Singapore, Singapore, Singapore; University of Bahrain, BAHRAIN

## Abstract

**Objectives:**

This study establishes age- and sex-specific reference values for fat mass index (FMI), lean mass index (LMI), appendicular LMI (aLMI), and body fat distribution indices including Android/Gynoid % fat ratio and Trunk/Limb % fat ratio in multi-ethnic Singaporean adults.

**Methods:**

A population-based cross-sectional study using dual-energy X-ray absorptiometry (Hologic Discovery Wi) was carried out to measure whole body and regional fat and lean mass in community-dwelling adults. A total of 537 adults (57.5% women), aged from 21 to 90 years, were recruited from the large north-eastern residential town of Yishun. Age- and sex-specific percentile reference values were generated for FMI, LMI, aLMI, Android/Gynoid % fat ratio and Trunk/Limb % fat ratio using the Lambda–Mu–Sigma method. The relationship between the parameters and age were assessed through the Pearson’s correlation coefficient.

**Results:**

All parameters demonstrated significant correlation with age (*p* < 0.05) for both men and women, except for LMI in women, with the strength of *r* ranging from 0.12 (weak correlation) to 0.54 (strong correlation). LMI (*r* = −0.45) and appendicular LMI (*r* = −0.54) were negatively associated with age in men while none (*r* = −0.06) to weak correlation (*r* = −0.14) were shown in women for the same parameters respectively. The Android/Gynoid % fat ratio and Trunk/Limb % fat ratio were positively related to age for both men (*r* = 0.37 & 0.43, *p* < 0.001) and women (*r* = 0.52 & 0.48, *p* < 0.001).

**Conclusion:**

We have established DXA-based body composition reference data for the Singapore adult population. These reference data will be particularly useful in geriatric, obesity and oncology clinics, enabling the prescription of appropriate therapy to individuals at risk of morbidity from unfavorable body composition phenotypes. It also adds on to the limited reference database on Southeast Asian body composition.

## Introduction

Body composition (BC) is closely related to health and includes parameters that define diseases/conditions such as sarcopenia, obesity, and low bone mass. These conditions in turn predict other diseases or adverse health, e.g. diabetes, cardio/cerebro-vascular diseases, cancers, fractures and functional disability that increase healthcare cost, reduce health span and lower quality of life [1–8]. While body mass index (BMI, total mass/height^2^), an anthropometric index, has been widely used in research and clinically as an indicator of adiposity, it is only a surrogate measure for obesity since it cannot differentiate between lean and fat mass [9–11]. BMI has also been used clinically to determine underweight and overweight status. Nevertheless, body composition is made up of components including muscle, fat, bone and water, hence expressing it using body weight relative to height meant that individuals with same BMI can have significantly different compositions, which can lead to the misclassification of persons carrying more weight in lean tissues as someone with excess body fat (high BMI) [12]. To overcome this limitation, more specific BC measures are necessary.

Dual X-ray absorptiometry (DXA), a non-invasive imaging modality that utilizes very low dose X-rays with two distinct energy peaks to image the soft tissue and bones of the human body, is considered the gold standard technique in the assessment of BC due to its precision and accuracy. It is capable of providing three BC measurements, namely: fat mass (FM), lean mass (LM) and bone mineral density (BMD), regionally and for the whole-body [13]. Although FM and LM measurements can accurately reflect body composition, it does not take a person’s height into account which in turn limit the comparisons between individuals, and therefore, the use of height adjusted fat mass index (FMI) and lean mass index (LMI) were proposed [14]. FMI was found to be more predictive of metabolic syndrome [11, 15] compared to BMI and is a better screening tool for obesity-related diseases including cardiovascular diseases, hypertension, stroke, type 2 diabetes mellitus, and some cancer types [16–21], while appendicular LMI (aLMI) is a key component for sarcopenia diagnosis [22–24]. Furthermore, the association between obesity and cardiovascular, as well as metabolic diseases is dependent on body fat distribution rather than total fat mass [25].

BC varies with age, sex and ethnicity, and so reference values should be population-specific and consider age, sex, and ethnicity [26, 27]. The National Health and Nutrition Examination Survey (NHANES) dataset, which is the most commonly used reference for body composition were developed from a cohort across the United States and provides reference BC values for Non-Hispanic Whites, Non-Hispanic Blacks and Mexican Americans. Therefore its application to Asians may not be appropriate [28, 29]. While there have been many reference value developed for the Western population [30–33], only a limited number were established for diverse Asian populations [34–37]. To our knowledge, a body composition reference database for multi-ethnic population in Singapore has yet to be established. Our study provides age- and sex-specific reference values for FMI, LMI, aLMI, and body fat indices (Trunk/Limb % fat ratio and Android/Gynoid % fat ratio) in a multi-ethnic Singaporean adult population using DXA. This enables meaningful interpretation of an individual’s BC result, giving an indication of his/her BC status relative to the average community dweller of the same age and sex.

## Methods

### Study design and participants

Community-dwelling adults aged ≥21 years old from Yishun, a large north-eastern residential town in Singapore comprising population of 220,320 (49.4% men), with 12.2% aged ≥65 years, were invited to participate in this study [38]. This is similar to the overall Singapore residential population consisting of 4.02 million (48.9% men), with 14.4% aged ≥65 years [38].

Recruitment within the Yishun region was conducted in two phases: between 1) October 2017 and February 2019; and 2) March and November 2019. A two-stage random sampling method was utilized in the first phase with 50% of all the housing blocks being randomly selected, followed by a random invitation of 20% of the units from those blocks to participate in the study. In the second phase, to increase the recruitment yield, 50% of the remaining housing blocks were randomly selected, and all the units within those blocks were invited to participate in the study. To achieve a representative sample of approximately 300 men and 300 women, a random sampling strategy was performed by filling quotas of 20–40 participants in each sex and age group (based on 10-year age groups between 21 and 60 years old; 5-year age-groups after 60 years old). This approach fulfilled the conventional recommendation of a minimum sample size of 30 per age-group for normative measures [39].

Door-to-door recruitment were conducted with up to three eligible members per household unit invited to participate in the study. Units with no response during the first home visit were contacted again at a different time of day on a later date. Recruitment of older individuals aged above 75 years old were extended to surrounding community including four senior activity centres. Exclusion criteria for recruitment include individuals with disabilities, injuries, fractures, or surgeries that affected function, neuromuscular, neurological, and cognitive impairments, or more than five poorly controlled comorbidities. Women who are pregnant or planning for pregnancy were also excluded. Ethics approval was obtained from the National Healthcare Group DSRB (2017/00212), in accordance with the relevant guidelines and regulations by the Declaration of Helsinki and the ethical principles in the Belmont Report. All participants gave written informed consent to participate in the study.

### Data collection

Participants self-reported their medical conditions and comorbidities. All assessments were based on standardised protocols and administered by trained researchers at the Geriatric Education & Research Institute Lab on Yishun Health Campus, mostly within one visit.

#### Anthropometry

Body weight and height were measured using an electronic scale and stadiometer, respectively (SECA, Hamburg, Germany). Body mass index was calculated as body weight (kg, measured to the nearest 0.1 kg) divided by height (m, measured to the nearest millimetre) squared.

#### Body composition measurement

Whole body scans were conducted using Hologic Discovery Wi (Hologic, Marlborough, MA, USA) Dual X-ray absorptiometry (DXA) scanner. The scan was performed by experienced radiography technologists (six technologists with each having experience of 3–6 years), using standardized protocol recommended by the manufacturer (Hologic). Prior to the examination, participants were asked to remove all garments and removable metal objects such as jewellery and watches and to change into a standard hospital gown that is free from zippers and buttons that would potentially interfere with the scan. Quality check and cross calibrations were regularly performed before each scanning session. The body composition parameters were analyzed using the Hologic APEX analysis software [40]. Due to the DXA scan table limitations, participants who were more than 136 kilograms or greater than 1.96 metres in height were excluded.

For this analysis, whole body (including the head) and regional (including limbs, trunk, android, and gynoid) FM (in kg) and LM (in kg) were obtained from the DXA dataset. From these measurements, the following parameters were calculated: FMI (total fat mass/height^2^); LMI (total lean mass/height^2^); appendicular FMI (aFMI, appendicular fat mass/height^2^); aLMI (appendicular lean mass/height^2^); Trunk/Limb % fat ratio; Android/Gynoid % fat ratio; and % FM (total body fat mass/weight × 100%). The appendicular region refers to both upper and lower limbs. Trunk/Limb % fat ratio and Android/Gynoid % fat ratio were included as indices of body fat distribution.

### Statistical analysis

All statistical analyses were performed using R V.3.6.2 (R Foundation for statistical computing, Vienna, Austria). For the descriptive analysis of participants’ characteristics, continuous variables were presented using means and SDs after dataset was stratified by sex and divided into the following age groups: 21–30; 31–40; 41–50; 51–60; 61–65; 66–70; 71–75; 76–80; > 80 years. Participant characteristics within each age group were analysed using independent samples t-test to assess potential differences between men and women. Age- and sex-specific reference values for body composition parameters were generated using the Lamda Mu Sigma (LMS) method of Cole and Green, as an extension of the normal distribution that adjusts for skewness, embedded in Generalized Additive Models for Location Scale and Shape (GAMLSS package version 5.2.0), for FMI, LMI, aLMI, Trunk/Limb % fat ratio and Android/Gynoid % fat ratio. The LMS method is equivalent to Box-Cox Cole and Green distribution (BCCG), and BCCG parameters (μ, σ, υ) are the approximate median, coefficient of variation and skewness parameters of the distribution of the response variable [41, 42]. That is, μ controls the location, σ controls the scale and υ controls the skewness of the distribution with age [41]. Generalized Akaike information criterion (GAIC) was used to determine the appropriate degrees of freedom [43]. The final GAMLSS model parameters were used to construct centiles tables for each sex and anthropometric measure of interest. Sex-stratified natural regression spline curves were also generated with 4 knots for FMI, LMI, aLMI, Trunk/Limb % fat ratio and Android/Gynoid % fat ratio. The relationships between each parameter and age were assessed separately in men and women using Pearson’s correlation coefficient (*r*). Correlation coefficient can range from -1.0 to +1.0 where the sign (+/-) indicates the direction and the numeric value representing the strength of the relationship. In terms of strength, a value of 0.5 or greater represented strong correlation; a value of 0.3 to less than 0.5, moderate correlation; and a value of 0.1 to less than 0.3, weak correlation [44]. The level of significance was set at *p* < 0.05.

## Results

The overall response rate was 39.0%. A total of 537 participants (57.5% women) aged from 21 to 90 years were recruited with the ethnic composition of 81.6% Chinese, 8.9% Malay, 6.7% Indians, and 2.8% others.

Table 1 showed participants’ characteristics. Within the same age group, significant differences (*p* < 0.05) were shown between men and women for all anthropometric characteristics and body composition parameters except for BMI (for age group 41–50; 51–60; 61–65; 66–70; 71–75; 76–80; and >80), FM (for age group 21–30; 31–40; 41–50; 71–75; and >80), FMI (for age group 21–30 and 31–40) and aFMI (for age group 21–30; and 31–40) (Table 1). Men had higher indices of body fat distribution (Android/Gynoid % fat ratio and Trunk/Limb % fat ratio) and all parameters associated with lean mass (LM, %LM, LMI, and aLMI) while women had higher indices in parameters associated with fat mass (FM, %FM, FMI, and aFMI) (*p* < 0.05; Table 1).

**Table 1:** Mean (SD) of baseline participant characteristics stratified by age groups for overall, male and females respectively.

	Age groups								
	21–30	31–40	41–50	51–60	61–65	66–70	71–75	76–80	>80
**Total**									
n	60	60	59	60	60	60	58	60	60
Age (years)	25.1 (2.8)	35.9 (2.9)	45.7 (2.8)	55.9 (3)	63.1 (1.4)	68 (1.5)	72.7 (1.6)	77.9 (1.4)	83.3 (2.2)
Height (m)	1.66 (0.09)	1.64 (0.08)	1.61 (0.08)	1.61 (0.09)	1.61 (0.08)	1.59 (0.07)	1.59 (0.08)	1.56 (0.08)	1.53 (0.09)
Weight (kg)	68.3 (20.8)	69.9 (18.8)	67.9 (13.8)	66.9 (13.8)	62.4 (9.1)	62 (9.5)	59.6 (10.2)	59.9 (9.5)	56.2 (10.6)
BMI (kg/m^2^)	24.6 (6.8)	26 (5.9)	26.2 (4.1)	25.7 (4.7)	24.2 (3.2)	24.6 (3.2)	23.5 (3.5)	24.4 (3.3)	23.9 (4)
FM (kg)	22.9 (11)	23.6 (9.9)	24.3 (6.9)	23.4 (7.4)	21.6 (5.9)	22.1 (5.8)	20.5 (5.5)	21.5 (5.9)	20.4 (5.9)
LM (kg)	41.5 (11.5)	42.4 (10.8)	40 (8.8)	40.3 (9.4)	37.5 (7)	36.4 (6.5)	35.6 (6.7)	34.9 (6.3)	32.6 (6.8)
%FM	33.6 (8.1)	34.3 (7.3)	36.4 (6.1)	35.6 (7.3)	35.2 (7.7)	36.4 (7)	35.1 (6.8)	36.5 (7.4)	36.9 (7.1)
%LM	62.8 (7.8)	62.3 (7.1)	60.3 (6)	61.5 (7)	61.5 (7.5)	60.2 (6.8)	61.3 (6.5)	59.9 (7.1)	59.6 (6.7)
FMI (kg/m^2^)	8.4 (4.1)	8.8 (3.6)	9.4 (2.6)	9.1 (3.1)	8.5 (2.7)	8.9 (2.5)	8.2 (2.3)	8.9 (2.7)	8.8 (2.8)
LMI (kg/m^2^)	14.9 (3.3)	15.7 (3)	15.4 (2.4)	15.4 (2.6)	14.5 (1.9)	14.4 (1.7)	14 (1.9)	14.2 (1.7)	13.8 (1.9)
Appendicular FMI (kg/m^2^)	4 (1.9)	4.2 (1.7)	4.4 (1.4)	4.2 (1.8)	3.8 (1.4)	3.8 (1.3)	3.4 (1.1)	3.8 (1.4)	3.6 (1.3)
Appendicular LMI (kg/m^2^)	6.5 (1.7)	6.8 (1.6)	6.6 (1.3)	6.6 (1.5)	6.1 (0.9)	6 (0.9)	5.8 (1)	5.8 (0.9)	5.6 (0.9)
Android/Gynoid % fat ratio	0.43 (0.16)	0.48 (0.14)	0.53 (0.15)	0.55 (0.15)	0.6 (0.18)	0.62 (0.16)	0.63 (0.2)	0.63 (0.16)	0.64 (0.16)
Trunk/Limbs % fat ratio	0.99 (0.22)	1.04 (0.22)	1.1 (0.26)	1.1 (0.27)	1.2 (0.3)	1.28 (0.26)	1.34 (0.36)	1.29 (0.25)	1.36 (0.29)
**Male**									
n	28	26	20	22	29	25	29	26	23
Age (years)	25.1 (2.8)	35.9 (2.9)	45.8 (2.5)	57 (2.5)*	63.1 (1.4)	68.4 (1.4)	72.9 (1.7)	77.9 (1.3)	83.7 (2.3)
Height (m)	1.73 (0.07)***	1.70 (0.05)***	1.68 (0.06)***	1.69 (0.07)***	1.66 (0.06)***	1.65 (0.05)***	1.65 (0.06)***	1.63 (0.07)***	1.62 (0.07)***
Weight (kg)	80.4 (22.4)***	81.2 (20.0)***	76.8 (13.4)***	73.5 (10.9)**	66.2 (8.0)**	65.9 (10.7)*	65.4 (8.5)***	63.0 (10.3)*	61.6 (11.4)**
BMI (kg/m^2^)	27.1 (8.2)*	28.0 (6.7)*	27.2 (3.8)	25.7 (3.2)	24.0 (2.9)	24.1 (3.4)	24.2 (3.2)	23.7 (3.0)	23.5 (4.1)
FM (kg)	24.6 (13.9)	24.2 (12.3)	23.6 (7.1)	20.2 (5.0)**	19.1 (4.9)**	19.6 (6.1)**	20.1 (5.7)	18.9 (5.7)**	18.9 (6.0)
LM (kg)	51.2 (9.0)***	52.1 (8.4)***	49.0 (6.9)***	49.3 (6.7)***	43.4 (4.5)***	42.2 (5.2)***	41.3 (3.4)***	40.0 (5.1)***	38.9 (6.4)***
%FM	29.6 (8.1)***	29.7 (6.7)***	31.0 (4.8)***	28.0 (4.3)***	29.1 (5.1)***	30.0 (5.0)***	30.9 (5.8)***	30.3 (4.9)***	31.1 (5.4)***
%LM	66.9 (7.6)***	67.0 (6.4)***	65.9 (4.8)***	68.8 (4.2)***	67.4 (4.9)***	66.3 (4.7)***	65.3 (5.5)***	65.7 (4.8)***	65.1 (5.1)***
FMI (kg/m^2^)	8.4 (5.2)	8.3 (4.3)	8.3 (2.3)*	7.1 (1.7)***	6.9 (1.8)***	7.2 (2.1)***	7.4 (2.1)*	7.1 (1.9)***	7.3 (2.3)***
LMI (kg/m^2^)	17.2 (3.0)***	18 (2.6)***	17.4 (1.9)***	17.2 (1.9)***	15.7 (1.5)***	15.4 (1.4)***	15.3 (1.4)***	15.1 (1.4)***	14.8 (2.0)**
Appendicular FMI (kg/m^2^)	3.8 (2.4)	3.7 (1.9)	3.5 (1.1)***	2.9 (0.7)***	2.7 (0.8)***	2.8 (0.9)***	2.9 (0.8)***	2.8 (0.8)***	2.8 (0.9)***
Appendicular LMI (kg/m^2^)	7.9 (1.4)***	8.2 (1.3)***	7.7 (1.1)***	7.7 (1.1)***	6.7 (0.7)***	6.7 (0.7)***	6.5 (0.7)***	6.4 (0.7)***	6.2 (1.0)***
Android/Gynoid % fat ratio	0.52 (0.18)***	0.57 (0.11)***	0.67 (0.13)***	0.66 (0.11)***	0.71 (0.15)***	0.70 (0.15)***	0.71 (0.19)***	0.70 (0.18)**	0.69 (0.13)*
Trunk/Limbs % fat ratio	1.10 (0.24)***	1.17 (0.18)***	1.31 (0.23)***	1.29 (0.26)***	1.41 (0.25)***	1.45 (0.24)***	1.46 (0.34)**	1.37 (0.24)*	1.45 (0.21)
**Female**									
n	32	34	39	38	31	35	29	34	37
Age (years)	25.1 (2.8)	35.9 (3)	45.6 (3)	55.2 (3.1)*	63.1 (1.4)	67.8 (1.5)	72.5 (1.6)	77.9 (1.5)	83.1 (2.1)
Height (m)	1.60 (0.05)***	1.59 (0.06)***	1.57 (0.07)***	1.57 (0.06)***	1.55 (0.05)***	1.54 (0.05)***	1.53 (0.05)***	1.52 (0.05)***	1.48 (0.04)***
Weight (kg)	57.7 (11.7)***	61.3 (12.4)***	63.4 (11.7)***	63 (13.9)**	58.8 (8.7)**	59.3 (7.6)*	53.8 (8.4)***	57.5 (8.2)*	52.8 (8.6)**
BMI (kg/m^2^)	22.5 (4.5)*	24.4 (4.7)*	25.7 (4.3)	25.7 (5.5)	24.4 (3.6)	25.0 (3.0)	22.9 (3.7)	25.0 (3.5)	24.2 (4.0)
FM (kg)	21.5 (7.6)	23.2 (7.9)	24.7 (6.9)	25.2 (7.9)**	23.9 (5.9)**	23.9 (4.8)**	21.0 (5.2)	23.4 (5.4)**	21.2 (5.8)
LM (kg)	32.9 (4.6)***	34.9 (4.8)***	35.4 (5.4)***	35.1 (6.3)***	32.1 (3.7)***	32.2 (3.5)***	29.9 (3.6)***	31.0 (3.9)***	28.7 (3.4)***
%FM	37.0 (6.4)***	37.7 (5.7)***	39.2 (4.7)***	40.0 (4.5)***	40.9 (4.9)***	41.0 (4.1)***	39.3 (5.0)***	41.2 (5.1)***	40.5 (5.5)***
%LM	59.2 (6.0)***	58.7 (5.3)***	57.5 (4.4)***	57.2 (4.2)***	56.0 (4.7)***	55.9 (4.2)***	57.3 (4.7)***	55.4 (4.9)***	56.2 (5.2)***
FMI (kg/m^2^)	8.3 (2.9)	9.2 (3.0)	10.0 (2.7)*	10.3 (3.2)***	9.9 (2.5)***	10.1 (2.0)***	8.9 (2.3)*	10.2 (2.4)***	9.7 (2.7)***
LMI (kg/m^2^)	12.8 (1.8)***	13.9 (1.8)***	14.3 (1.9)***	14.3 (2.4)***	13.3 (1.4)***	13.6 (1.4)***	12.7 (1.5)***	13.5 (1.6)***	13.2 (1.6)**
Appendicular FMI (kg/m^2^)	4.2 (1.5)	4.5 (1.5)	4.8 (1.3)***	5.0 (1.7)***	4.7 (1.2)***	4.5 (1)***	3.9 (1.2)***	4.5 (1.4)***	4.1 (1.3)***
Appendicular LMI (kg/m^2^)	5.4 (0.9)***	5.8 (0.9)***	6.0 (1.0)***	6.0 (1.3)***	5.5 (0.7)***	5.6 (0.7)***	5.2 (0.7)***	5.4 (0.7)***	5.2 (0.7)***
Android/Gynoid % fat ratio	0.35 (0.08)***	0.40 (0.11)***	0.45 (0.10)***	0.49 (0.12)***	0.49 (0.13)***	0.56 (0.14)***	0.54 (0.17)***	0.58 (0.13)**	0.60 (0.17)*
Trunk/Limbs % fat ratio	0.90 (0.17)***	0.94 (0.19)***	0.99 (0.19)***	0.98 (0.20)***	1.01 (0.20)***	1.16 (0.19)***	1.22 (0.34)**	1.22 (0.24)*	1.31 (0.32)

*p<0.05

**p<0.01

***p<0.001 for significant sex effects within age group. n, sample size for each age group; BMI, body mass index; FM, fat mass; LM, lean mass; FMI, fat mass index; LMI, lean mass index.

**Fig 1:**
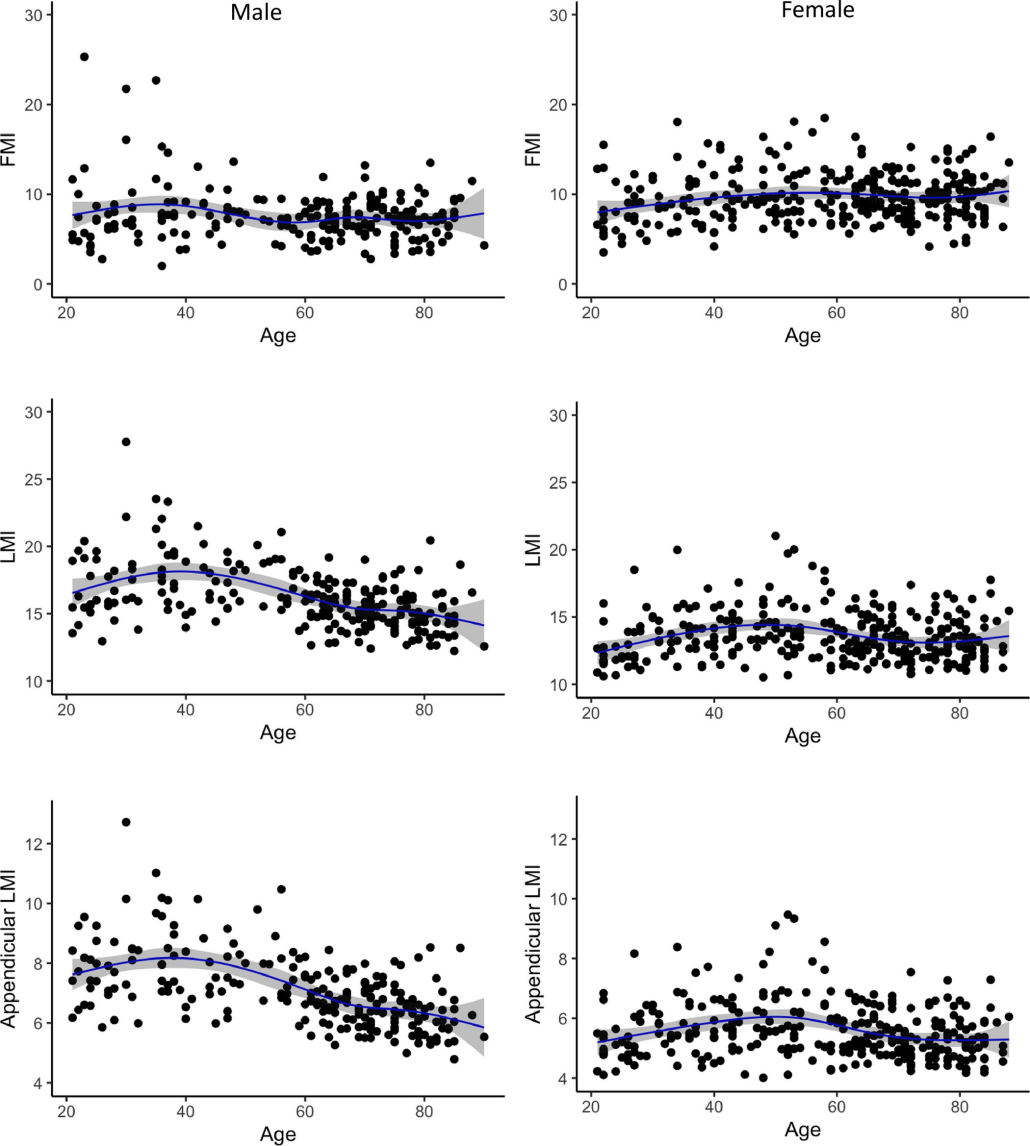
Natural spline regression by sex for FMI, LMI, and appendicular LMI in men and women aged 21–90 years. FMI, fat mass index; LMI, lean mass index.

**Fig 2:**
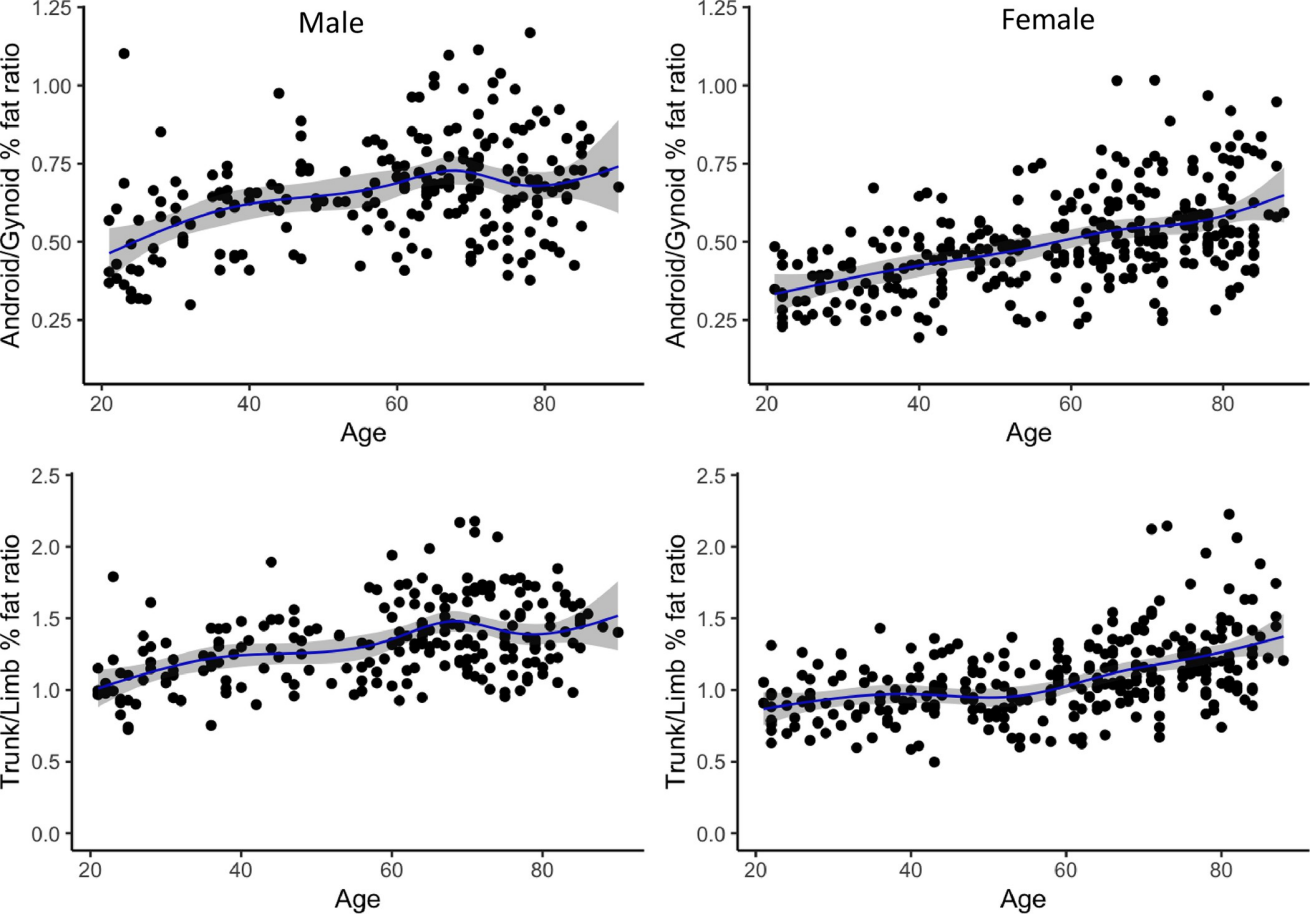
Natural spline regression by sex for Android/Gynoid % fat ratio and Trunk/Limb % fat ratio in men and women aged 21–90 years.

Using the LMS method, sex-stratified values for 3^rd^, 10^th^ 50^th^, 90^th^, and 97^th^ percentiles for individuals aged 21–90 years were generated for FMI, LMI, aLMI, Android/Gynoid % fat ratio and Trunk/Limb % fat ratio (Tables 2–6), with the age in each table representing the age point for the predicted centile LMS values generated using GAMLSS. The differences between the sexes were also demonstrated in the Spline regression plots where LMI, aLMI, Android/Gynoid % fat ratio and Trunk/Limb % fat ratio were consistently higher in men of all age groups compared to women (Figs 1 and 2). Significant correlation (*p* < 0.05) with age were shown for all variables for both men and women, except for LMI in women, with the strength of *r* ranging from 0.12 (weak correlation) to 0.54 (strong correlation) (Table 7). The LMI (*r* = −0.45, *p* < 0.001) and appendicular LMI (*r* = −0.54, *p* < 0.001) were negatively associated with age in general and decline started around 40 years of age in men (Table 7 and Fig 1). None (*r* = −0.06, *p* = 0.316) to weak correlation (*r* = −0.14, *p* = 0.012) were shown in women for the same parameters respectively (Table 7). The Android/Gynoid % fat ratio and Trunk/Limb % fat ratio were positively related to age for both men (*r* = 0.37 & 0.43, *p* < 0.001) and women (*r* = 0.52 & 0.48, *p* < 0.001) with the latter showing a steady increase with age (Table 7 and Fig 2).

**Table 2:** Sex-specific percentile values for fat mass index (FMI) in individuals aged 21–90 years.

		Male								Female						
				Percentile								Percentile				
Age (years)	L	M	S	3	10	50	90	97	L	M	S	3	10	50	90	97
25	-0.23	7.44	0.50	3.15	4.07	7.44	14.98	21.67	0.04	8.20	0.33	4.35	5.33	8.20	12.53	15.24
30	-0.10	7.41	0.46	3.22	4.16	7.41	13.67	18.46	-0.02	8.50	0.32	4.68	5.65	8.50	12.82	15.56
35	0.02	7.37	0.42	3.32	4.29	7.37	12.61	16.19	-0.08	8.78	0.31	4.99	5.96	8.78	13.08	15.83
40	0.13	7.33	0.38	3.48	4.45	7.33	11.73	14.47	-0.12	9.02	0.29	5.28	6.24	9.02	13.29	16.03
45	0.23	7.30	0.34	3.67	4.62	7.30	11.02	13.20	-0.15	9.24	0.28	5.52	6.48	9.24	13.43	16.12
50	0.33	7.26	0.31	3.83	4.77	7.26	10.50	12.31	-0.14	9.41	0.27	5.71	6.67	9.41	13.50	16.09
55	0.42	7.22	0.29	3.94	4.86	7.22	10.15	11.72	-0.09	9.53	0.27	5.84	6.81	9.53	13.48	15.92
60	0.49	7.19	0.27	3.97	4.89	7.19	9.93	11.37	0.01	9.61	0.26	5.89	6.89	9.61	13.40	15.64
65	0.56	7.15	0.27	3.92	4.86	7.15	9.82	11.19	0.15	9.66	0.25	5.88	6.92	9.66	13.27	15.32
70	0.62	7.11	0.27	3.82	4.78	7.11	9.79	11.14	0.31	9.69	0.25	5.82	6.91	9.69	13.15	15.03
75	0.67	7.08	0.28	3.67	4.68	7.08	9.79	11.16	0.48	9.72	0.25	5.73	6.88	9.72	13.07	14.82
80	0.71	7.04	0.29	3.50	4.55	7.04	9.82	11.21	0.64	9.76	0.25	5.62	6.85	9.76	13.03	14.67
85	0.75	7.01	0.30	3.31	4.42	7.01	9.86	11.28	0.78	9.81	0.25	5.51	6.82	9.81	13.01	14.57
90	0.79	6.97	0.32	3.10	4.26	6.97	9.92	11.37	0.93	9.86	0.25	5.38	6.79	9.86	13.01	14.50

L (lambda), M (mu); S (sigma)

**Table 3:** Sex-specific percentile values for lean mass index (LMI) in individuals aged 21–90 years.

		Male								Female						
				Percentile								Percentile				
Age (years)	L	M	S	3	10	50	90	97	L	M	S	3	10	50	90	97
25	-1.45	16.9	0.14	13.5	14.4	16.9	20.8	23.6	-1.64	12.6	0.12	10.5	11.1	12.6	15.0	16.6
30	-1.63	17.2	0.13	13.9	14.8	17.2	21.0	23.8	-1.66	13.2	0.12	10.9	11.5	13.2	15.6	17.3
35	-1.50	17.4	0.13	14.1	15.0	17.4	21.0	23.4	-1.67	13.5	0.12	11.2	11.8	13.5	16.2	18.0
40	-1.07	17.4	0.12	14.2	15.1	17.4	20.7	22.7	-1.69	13.8	0.12	11.4	12.0	13.8	16.5	18.4
45	-0.52	17.3	0.12	14.1	15.0	17.3	20.3	21.9	-1.71	13.9	0.12	11.4	12.1	13.9	16.6	18.6
50	0.02	17.1	0.11	13.9	14.9	17.1	19.7	21.1	-1.73	13.8	0.12	11.4	12.1	13.8	16.6	18.5
55	0.48	16.8	0.11	13.6	14.6	16.8	19.1	20.3	-1.75	13.7	0.12	11.3	12.0	13.7	16.4	18.3
60	0.75	16.3	0.10	13.3	14.2	16.3	18.5	19.5	-1.77	13.5	0.12	11.2	11.8	13.5	16.1	17.9
65	0.61	15.8	0.10	13.0	13.9	15.8	17.9	18.9	-1.78	13.3	0.12	11.1	11.7	13.3	15.8	17.5
70	-0.04	15.4	0.10	12.8	13.6	15.4	17.4	18.5	-1.80	13.1	0.11	11.0	11.5	13.1	15.5	17.1
75	-1.06	15.0	0.10	12.7	13.4	15.0	17.1	18.3	-1.82	13.0	0.11	10.9	11.5	13.0	15.3	16.9
80	-2.29	14.6	0.09	12.6	13.1	14.6	16.8	18.3	-1.84	13.0	0.11	10.9	11.5	13.0	15.2	16.7
85	-3.55	14.1	0.09	12.3	12.8	14.1	16.5	18.5	-1.86	13.0	0.11	11.0	11.5	13.0	15.2	16.7
90	-4.83	13.7	0.09	12.0	12.4	13.7	16.1	18.6	-1.88	13.0	0.10	11.0	11.5	13.0	15.1	16.6

L (lambda), M (mu); S (sigma)

**Table 4:** Sex-specific percentile values for appendicular lean mass index (aLMI) in individuals aged 21–90 years.

		Male								Female						
				Percentile								Percentile				
Age (years)	L	M	S	3	10	50	90	97	L	M	S	3	10	50	90	97
25	-0.69	7.81	0.16	5.95	6.45	7.81	9.73	10.93	-0.91	5.28	0.14	4.15	4.45	5.28	6.45	7.18
30	-0.75	7.84	0.15	6.04	6.53	7.84	9.70	10.86	-0.95	5.45	0.15	4.26	4.58	5.45	6.73	7.55
35	-0.80	7.84	0.15	6.10	6.57	7.84	9.63	10.74	-1.00	5.59	0.16	4.33	4.67	5.59	6.98	7.90
40	-0.86	7.78	0.14	6.11	6.57	7.78	9.49	10.55	-1.04	5.68	0.16	4.36	4.71	5.68	7.18	8.20
45	-0.91	7.67	0.14	6.08	6.51	7.67	9.28	10.28	-1.09	5.72	0.17	4.36	4.72	5.72	7.32	8.43
50	-0.97	7.50	0.13	6.01	6.42	7.50	9.02	9.96	-1.13	5.71	0.17	4.35	4.7	5.71	7.34	8.49
55	-1.02	7.29	0.13	5.88	6.27	7.29	8.71	9.59	-1.17	5.65	0.16	4.35	4.69	5.65	7.19	8.28
60	-1.08	7.03	0.12	5.72	6.08	7.03	8.36	9.17	-1.22	5.56	0.15	4.36	4.68	5.56	6.92	7.86
65	-1.13	6.77	0.12	5.54	5.88	6.77	8.01	8.78	-1.26	5.45	0.14	4.36	4.65	5.45	6.64	7.42
70	-1.19	6.55	0.12	5.39	5.70	6.55	7.72	8.44	-1.30	5.33	0.13	4.32	4.59	5.33	6.41	7.11
75	-1.24	6.35	0.11	5.24	5.55	6.35	7.46	8.15	-1.35	5.23	0.12	4.27	4.53	5.23	6.26	6.93
80	-1.30	6.15	0.11	5.10	5.39	6.15	7.22	7.88	-1.39	5.16	0.12	4.22	4.47	5.16	6.17	6.84
85	-1.35	5.96	0.11	4.96	5.23	5.96	6.98	7.61	-1.43	5.11	0.12	4.17	4.42	5.11	6.12	6.79
90	-1.41	5.77	0.11	4.82	5.08	5.77	6.75	7.36	-1.48	5.06	0.13	4.14	4.38	5.06	6.08	6.76

L (lambda), M (mu); S (sigma)

**Table 5:** Sex-specific percentile values for Android/Gynoid % fat ratio in individuals aged 21–90 years.

		Male								Female						
				Percentile								Percentile				
Age (years)	L	M	S	3	10	50	90	97	L	M	S	3	10	50	90	97
25	-0.55	0.48	0.26	0.31	0.35	0.48	0.7	0.86	0.69	0.35	0.24	0.21	0.25	0.35	0.47	0.52
30	0.18	0.53	0.24	0.33	0.38	0.53	0.71	0.81	0.65	0.37	0.24	0.22	0.27	0.37	0.50	0.56
35	0.65	0.57	0.22	0.35	0.42	0.57	0.73	0.82	0.62	0.39	0.24	0.23	0.28	0.39	0.52	0.59
40	0.82	0.61	0.20	0.38	0.45	0.61	0.77	0.84	0.59	0.41	0.24	0.24	0.29	0.41	0.55	0.62
45	0.82	0.64	0.19	0.41	0.48	0.64	0.8	0.88	0.56	0.44	0.25	0.25	0.31	0.44	0.58	0.66
50	0.79	0.66	0.19	0.43	0.50	0.66	0.82	0.90	0.53	0.46	0.25	0.27	0.32	0.46	0.61	0.69
55	0.71	0.67	0.19	0.44	0.51	0.67	0.85	0.93	0.49	0.48	0.25	0.28	0.33	0.48	0.64	0.73
60	0.55	0.69	0.20	0.45	0.52	0.69	0.87	0.97	0.46	0.50	0.25	0.29	0.35	0.50	0.67	0.76
65	0.36	0.69	0.21	0.45	0.52	0.69	0.9	1.01	0.43	0.52	0.26	0.30	0.36	0.52	0.70	0.80
70	0.29	0.70	0.23	0.44	0.51	0.70	0.92	1.04	0.40	0.54	0.26	0.31	0.38	0.54	0.73	0.84
75	0.40	0.69	0.23	0.43	0.51	0.69	0.92	1.04	0.37	0.56	0.26	0.32	0.39	0.56	0.76	0.88
80	0.66	0.69	0.23	0.42	0.50	0.69	0.9	1.01	0.34	0.58	0.26	0.34	0.40	0.58	0.80	0.92
85	1.03	0.69	0.22	0.40	0.49	0.69	0.88	0.96	0.30	0.60	0.27	0.35	0.42	0.60	0.83	0.95
90	1.42	0.68	0.21	0.39	0.49	0.68	0.85	0.93	0.27	0.62	0.27	0.36	0.43	0.62	0.86	0.99

L (lambda), M (mu); S (sigma)

**Table 6:** Sex-specific percentile values for Trunk/Limb % fat ratio in individuals aged 21–90 years.

		Male								Female						
				Percentile								Percentile				
Age (years)	L	M	S	3	10	50	90	97	L	M	S	3	10	50	90	97
25	-0.29	1.06	0.18	0.77	0.85	1.06	1.36	1.53	-0.11	0.89	0.19	0.63	0.7	0.89	1.14	1.28
30	-0.26	1.11	0.18	0.80	0.89	1.11	1.42	1.59	0.18	0.91	0.19	0.62	0.71	0.91	1.15	1.29
35	-0.22	1.16	0.18	0.83	0.92	1.16	1.47	1.66	0.44	0.92	0.19	0.62	0.71	0.92	1.17	1.30
40	-0.18	1.21	0.18	0.87	0.96	1.21	1.53	1.71	0.67	0.94	0.20	0.62	0.72	0.94	1.19	1.31
45	-0.14	1.25	0.18	0.89	0.99	1.25	1.58	1.77	0.83	0.96	0.20	0.62	0.72	0.96	1.21	1.33
50	-0.10	1.28	0.18	0.92	1.02	1.28	1.62	1.82	0.89	0.98	0.20	0.62	0.73	0.98	1.23	1.35
55	-0.06	1.32	0.18	0.94	1.05	1.32	1.66	1.86	0.84	1.00	0.20	0.64	0.75	1.00	1.27	1.39
60	-0.03	1.35	0.18	0.96	1.07	1.35	1.70	1.90	0.67	1.04	0.20	0.67	0.78	1.04	1.32	1.46
65	0.01	1.38	0.18	0.98	1.09	1.38	1.73	1.93	0.38	1.08	0.20	0.71	0.82	1.08	1.38	1.54
70	0.05	1.40	0.18	0.99	1.11	1.40	1.75	1.95	0.03	1.12	0.21	0.76	0.86	1.12	1.46	1.65
75	0.09	1.41	0.18	1.00	1.12	1.41	1.77	1.96	-0.30	1.17	0.21	0.81	0.90	1.17	1.54	1.77
80	0.13	1.42	0.18	1.01	1.13	1.42	1.78	1.97	-0.61	1.21	0.21	0.85	0.95	1.21	1.63	1.90
85	0.17	1.43	0.18	1.01	1.13	1.43	1.79	1.98	-0.90	1.26	0.21	0.90	0.99	1.26	1.72	2.06
90	0.20	1.44	0.18	1.02	1.14	1.44	1.80	1.99	-1.18	1.31	0.21	0.94	1.04	1.31	1.82	2.25

L (lambda), M (mu); S (sigma)

**Table 7:** List of reference values generated and correlation with age (years) in men and women respectively.

	Men (n = 228)		Women (n = 309)	
Variables	Pearson Correlation (95%CI)	*P* value	Pearson Correlation (95%CI)	*P* value
FMI (kg/m^2^)	-0.16 (-0.28, -0.03)	0.014	0.12 (0.01, 0.23)	0.033
LMI (kg/m^2^)	-0.45 (-0.55, -0.34)	<0.001	-0.06 (-0.17, 0.05)	0.316
Appendicular LMI (kg/m^2^)	-0.54 (-0.63, -0.44)	<0.001	-0.14 (-0.25, -0.03)	0.012
Android/Gynoid % fat ratio	0.37 (0.24, 0.48)	<0.001	0.52 (0.43, 0.60)	<0.001
Trunk/Limbs % fat ratio	0.43 (0.32, 0.53)	<0.001	0.48 (0.39, 0.56)	<0.001

n, sample size; FMI, fat mass index; LMI, lean mass index.

## Discussion

While it has been long established that BC reference values should be population-specific and take age, sex, and ethnicity into consideration [26, 27], the lack of local reference values has hitherto limited the adoption of BC screening in clinical practice. To our knowledge, this is the first study presenting age- and sex-specific reference values for FMI, LMI, aLMI, Android/Gynoid % fat ratio, and Trunk/Limb % fat ratio, based on a nationally and ethnically representative data set that were acquired using established DXA technology, equipment, and procedure.

### FMI

FMI, which is calculated as the total body fat mass with height^2^ adjustment, is a direct adiposity measurement of the human body. This parameter has been proposed as a measure of abnormal fat mass level (low/excessive) and advocated for use in diagnosing clinical obesity although the appropriate cut-off point to diagnose obesity is still an ongoing debate [28, 45] FMI was shown to increase with age in both sexes from the Chinese and NHANES data [28, 34]. A Korean study on reference value demonstrated FMI trend amongst women similar to that of NHANES, however with no relationship was found in Korean men [36]. Interestingly, data from our study sample revealed a weakly correlated decrease in FMI with age, an effect in men that is opposite to the NHANES and Chinese data while women in our study are found to be consistent with previously reported trend. It has been shown that consumption of 113 grams of meat for five times a week helps in the prevention of sarcopenia [46]. Apart from diet, the contrasting evidence may also be attributed to differences in lifestyle between populations and future studies should examine this.

### LMI and aLMI

aLMI has been widely used in sarcopenia diagnosis, which is a condition characterized by gradual and general loss of muscle mass and strength, and have been shown to be predict functional disability in older adults [23, 24, 47, 48]. Our data demonstrated significant sex differences in lean mass parameters, with men having greater LMI and aLMI for all age groups, supporting the need for sex-specific reference value. Consistent with the Chinese study, the LMI and aLMI for men in the present study were also negatively correlated with age, however with a decline observed from around 40 years of age instead of the fifth decade [34]. The same study found no relationship in both parameters with age in women, and while LMI in our work similarly showed no relationship, a weak negative correlation were demonstrated in aLMI. These findings are consistent with previous studies on Asian populations where men were shown to be more likely to develop sarcopenia compared to women [49, 50].

### Body fat distribution

Body fat distribution has been shown to be a better predictor of health risk compared to total fat mass and therefore, Trunk/Limb % fat ratio and Android/Gynoid % fat ratio were included in our analysis [51, 52]. These indices were postulated to play a part in defining metabolic syndrome or lipodystrophy [53, 54]. Android adipose deposition was found to be associated with higher risk of cardiovascular and metabolic diseases, while fat accumulated around the gynoid is related to decreased risk of metabolic diseases and can potentially provide protection against harmful health effects in both sexes [51, 52, 55]. A linear relationship between sarcopenia and adiposity had also been suggested by another study [56].

From our dataset, there are indications of overall central fat accumulation with age in both sexes with the mean Android/Gynoid % fat ratio and Trunk/Limb % fat ratio increasing with age for the entire lifespan, except for Android/Gynoid % fat ratio in men which increased until 70 years of age. The men exhibited significantly higher central adiposity (Trunk/Limb % fat ratio, Android/Gynoid % fat ratio) compared to the women of all age groups although the latter have greater total body fat (% FM) in comparison to men. While the general trends are in line with previous studies [28, 30, 33, 36], other work have demonstrated higher central adiposity in Asian ethnicity compared to the Caucasian populations [57, 58]. Further work can elucidate the relationship between ethnicity, sex and central adiposity.

### Strengths, limitations, and future works

To our knowledge, this is the first study to publish sex- and age-specific BC reference data in Singaporean adults. Although with a modest sample size, the subjects were randomly recruited from a population which is similar to the overall Singapore residential population in terms of gender and older adults (≥65 years) proportion, thereby contributing to the limited reference database on Southeast Asian body composition. There are, however, several limitations in this study that should be acknowledged. Firstly, our overall modest sample size did not allow for ethnic-specific reference values to be generated. Secondly, the number of male participants in the age groups below 60 years was slightly fewer than anticipated. Thus the normative distribution limits of BC parameters for males below 60 years may not be an exact reflection of true population parameters [59]. Thirdly, this study only excluded subjects with more than five poorly controlled comorbidities, which may affect some of the reported reference values. Lastly, this cross-sectional data across adult age spectrum do not represent changes with age and was not intended so. Future population-based longitudinal study with larger sample size can address these limitations. In addition, there is a further urgent need to develop ethnic-specific reference values for a more accurate clinical application. Nevertheless, the reference values reported here should provide a better representation of local population compared to the commonly used NHANES dataset which is based on Western population.

## Conclusion

Our study presents the sex- and age-specific reference values in FMI, LMI, aLMI, and indices for body fat distribution, including Android/Gynoid % fat ratio and Trunk/Limb % fat ratio using DXA in multi-ethnic Singaporean adults. The importance of developing local reference values specific to our population is highlighted in this study. These reference data should prove useful for stratification of an individual’s BC result relative to persons of the same age and sex, and add on to the limited reference database on Southeast Asian body composition. This can inform future studies related to obesity, nutrition, and sarcopenia.

## Supporting information

S1 Dataset (CSV)
